# Pressure Ulcer Management Virtual Reality Simulation (PU-VRSim) for Novice Nurses: Mixed Methods Study

**DOI:** 10.2196/53165

**Published:** 2024-06-24

**Authors:** Soo Youn Jung, Kyoung Ja Moon

**Affiliations:** 1 College of Nursing Keimyung University Daegu Republic of Korea

**Keywords:** virtual reality, nursing, simulation, virtual training, pressure ulcer, simulation training, nurse, clinician, health care worker, ulcer, hospital, health care center, PU-VRSim, mixed methods study, health professional, medical education, training, games, gamification, learning, decubitus ulcer

## Abstract

**Background:**

Pressure ulcers (PUs) are a common and serious complication in patients who are immobile in health care settings. Nurses play a fundamental role in the prevention of PUs; however, novice nurses lack experience in clinical situations. Virtual reality (VR) is highly conducive to clinical- and procedure-focused training because it facilitates simulations.

**Objective:**

We aimed to explore the feasibility of a novel PU management VR simulation (PU-VRSim) program using a head-mounted display for novice nurses and to investigate how different types of learning materials (ie, VR or a video-based lecture) impact learning outcomes and experiences.

**Methods:**

PU-VRSim was created in the Unity 3D platform. This mixed methods pilot quasi-experimental study included 35 novice nurses categorized into the experimental (n=18) and control (n=17) groups. The PU-VRSim program was applied using VR in the experimental group, whereas the control group received a video-based lecture. The PU knowledge test, critical thinking disposition measurement tool, and Korean version of the General Self-Efficacy Scale were assessed before and after the intervention in both groups. After the intervention, the experimental group was further assessed using the Clinical Judgment Rubric and interviewed to evaluate their experience with PU-VRSim.

**Results:**

The results compared before and after the intervention showed significant improvements in PU knowledge in both the experimental group (*P*=.001) and control group (*P*=.005). There were no significant differences in self-efficacy and critical thinking in either group. The experimental group scored a mean of 3.23 (SD 0.44) points (accomplished) on clinical judgment, assessed using a 4-point scale. The experimental group interviews revealed that the VR simulation was realistic and helpful for learning about PU management.

**Conclusions:**

The results revealed that PU-VRSim could improve novice nurses’ learning of PU management in realistic environments. Further studies using VR for clinical training are recommended for novice nurses.

## Introduction

Pressure ulcers (PUs) refer to the skin damage caused by ischemia of the skin, subcutaneous fat, and muscles due to a continuous blood circulation disorder in a compressed body [1]. PUs result in a reduction of oxygen and nutrition delivered to the cells, which can contribute to the development of cancer and cardiovascular disease, ultimately amounting to high medical expenses [1]. The incidence of PUs is estimated at 12% in hospitals, representing a common but important health problem because it leads to high nursing burdens, increased medical costs, and mortality [2]. PUs are among the global health indicators and are included in the standard of nursing [3]. Most PUs are preventable by maintaining proper skin integration, and prevention is considered as important as treatment [3,4].

Nurses have a great responsibility in the well-being and safety of patients [5]. In addition, nurses are required to perform appropriate nursing for the prevention and management of PUs. However, they experience difficulties in performing clinical nursing and providing the necessary care to patients [6]. Novice nurses are those with less than 3 years of working experience based on the Benner novice-to-expert model; although they can recognize the basic order in nursing and take decisions, it is generally more difficult for novice nurses to establish priorities [7]. Particularly, novice nurses who have completed the regular curriculum do not have sufficient opportunities for practice during their training and thus experience difficulties in adapting to a new environment and changes in roles in the clinical field, leading to stress and anxiety [8,9]. Therefore, a program that can help novice nurses adapt to the clinical environment is needed.

Virtual reality (VR) is characterized by interaction, immersion, and imagination, and has been increasingly used in the curriculum for nursing with great potential for course development [10]. VR using a head-mounted display (HMD) provides the learning experience of communication between medical staff and patients, as well as simulations of standardized and controlled situations [11]. VR use has been easily accepted by learners in various medical environments and plays an essential role in improving their performance [11,12]. In nursing education, VR has been used in areas such as cardiopulmonary resuscitation, respiratory nursing, and delivery nursing, as well as for improving professional knowledge, clinical reasoning skills, and learning satisfaction [13,14]. In addition, as a learning method, VR meets the expectations and learning styles of the new generation of young learners [14,15].

In nursing education, teaching methods have shifted from traditional lecture-style education to simulation education [15]. Lecture-style education is effective in terms of knowledge transfer to novice nurses. However, there is a limit of this approach in improving nursing work skills in hospitals where various problems can occur [16]. Video-based education for novice nurses is a time-efficient and economically effective method owing to the heavy workload and lack of physical time; however, this format often lacks an appropriate feedback system [17]. Simulation education provides educational opportunities for clinical practice without putting patients or others at risk, and learners have the advantage of safely learning from experience [18]. VR is a representative technology for simulation education [15]. VR simulation can be used by novice nurses freely, which has been shown to improve their knowledge, critical thinking, and self-efficacy [13,18], thereby helping them transform into professional clinical nurses.

Simulation creates a learning environment in which learners can experience intervention and treatment in a safe manner, and various educational theories and structural models can be applied to achieve effective learning results [19].

The Analysis, Design, Development, Implementation, and Evaluation (ADDIE) instructional design model [20] is an effective and efficient development model based on the five steps of analysis, design, development, implementation, and evaluation. Kolb’s experiential learning theory [21] states that learning is achieved through the process of “active experimentation,” starting with a “concrete experience,” “reflective observation,” and “abstract conceptualization.” Through the concrete experience of simulation, learners make reflective observations and abstract conceptualizations by trying and practicing new techniques in a safe environment, and they perform active experimentation to understand the patient’s situation in an actual clinical environment and provide appropriate nursing practice.

This study aimed to develop a nursing PU management VR simulation program (PU-VRSim) and assess the feasibility of the novel virtual program for novice nurses. Toward this end, we applied the ADDIE instructional design model and Kolb’s experiential learning theory. The first objective was to assess the feasibility of implementing PU-VRSim for nursing education on PUs. The second objective was to compare the effects of the VR program and video-based lectures on PU knowledge, self-efficacy, and critical thinking, and confirm the level of clinical judgment and experience of participants after undergoing PU-VRSim. The main research questions included: (1) Is implementing PU-VRSim for nursing education feasible? (2) What is the effect of PU-VRSim compared with that of video-based lectures? and (3) What are the participants’ experiences with PU-VRSim?

## Methods

### Design

This study applied Kolb’s experiential learning theory [21] based on the ADDIE model [20], which is a model of instructional design that was used to develop PU-VRSim for preventing and managing PUs using VR in a nursing education program. This was a mixed methods, pilot quasi-experimental study [22] including nurses with less than 2 years of clinical experience to confirm the effectiveness of PU-VRSim. PU-VRSim was created in the Unity 3D platform (Unity Technologies). Participants experienced the program through an HMD and hand controllers (HTC Corporation, VIVE pro).

### Participants

**Figure 1:**
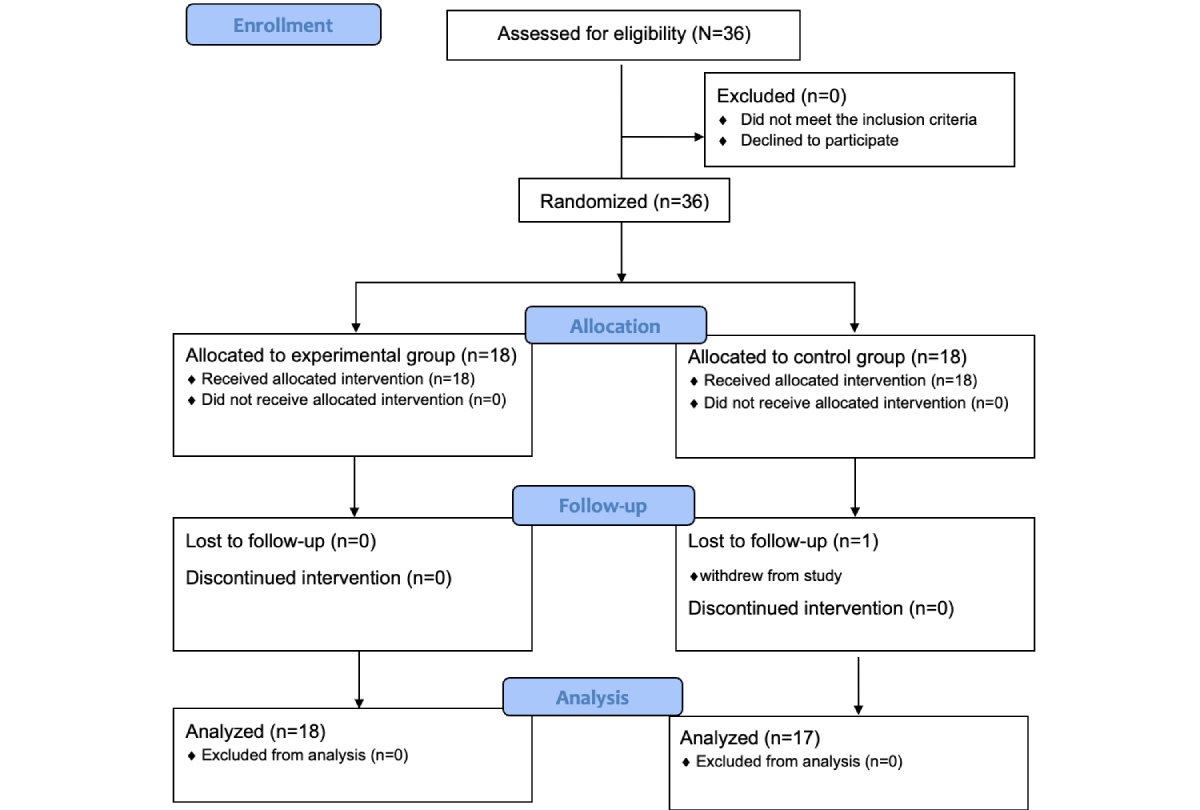
Flow diagram of participants’ enrollment, allocation, follow-up, and analysis.

For data collection, nurses with less than 2 years of clinical experience were notified of the purpose, period, conditions of participation, and benefits and disadvantages of participating in the study in the nurses’ community bulletin boards. Recruitment for the preintervention survey, intervention, and postintervention survey was conducted through convenience sampling. Participants were categorized into the two groups based on the work schedule of the novice nurses, and participants were blinded to their group allocation. A total of 35 participants were recruited voluntarily from October 10 to December 31, 2022, with 18 assigned to the experimental group and 17 assigned to the control group from January 1 to March 31, 2023. In both groups, one researcher conducted a one-on-one survey and measured general characteristics, PU knowledge, critical thinking, and self-efficacy using preliminary questionnaires, which were sent to the two groups before implementing the program. Regarding the intervention, the experimental group participated in the PU-VRSim program in the simulation room, whereas the control group participated in a video-based lecture on the prevention and care of PUs. After the program, PU knowledge, critical thinking skills, and self-efficacy were measured in both groups. The effectiveness of the program was further assessed with participants in the experimental group via interviews and the Lasater Clinical Judgment Rubric (LCJR) (Figure 1).

The sample size required to compare variables between groups with the *t* test was calculated using the G*Power 3.1 program according to the method of Polit and Sherman [23], using a significance level of α=.05, effect size (*f*) of 0.80, and power (1–β) of .90 [24]. Considering that the sample size satisfying the above conditions was at least 16 people per group, 36 participants were selected, including 18 in the experimental group and 18 in the control group, prior to data collection. One participant in the control group dropped out of the study. Finally, 35 participants were included in the analysis.

### Ethical Considerations

Data collection began after obtaining approval from the Institutional Review Board (40525-202204-HR-016-03) of Keimyung University in Daegu City for the protection of the research participants. The purpose of the study, procedures, guarantee of anonymity and confidentiality, and assurance that there are no consequences in case of withdrawal from the study were explained to the research participants, and they were allowed to respond to the questionnaire only when they agreed to participate in the research. The researchers conducted the preintervention survey, application of programs, and postintervention survey. All data collected during this study were anonymized. Participants were compensated for their contribution with a beverage coupon worth 10,000 KRW (~US $8) after the postintervention survey.

### Instruments

#### PU Knowledge

The Pieper–Zulkowski pressure ulcer knowledge test (PZ-PUKT), a PU knowledge tool developed by Pieper and Zulkowski [25] and modified and supplemented by Park [26], was used in this study. The PZ-PUKT comprises 39 questions, including 19, 9, and 11 questions on PU stage confirmation, wound assessment, and dressing methods, respectively. Each question was answered “yes,” “no,” or “don’t know,” with 1 point for correct answers and 0 points for incorrect answers. The total score ranges from 0 to 39, with higher scores indicating greater knowledge of PUs. The Cronbach α value was 0.80 and 0.70 in the studies by Pieper and Zulkowski [25] and Park [26], respectively, and was 0.69 in our study.

#### Critical Thinking

The critical thinking disposition measurement tool developed by Yun [27] and modified and supplemented by Shin et al [28] was used for evaluating the impact of the intervention on critical thinking skills. This tool comprises 27 questions divided into 7 subdomains: intellectual passion/curiosity (5 questions), prudence (4 questions), confidence (4 questions), systemicity (3 questions), intellectual fairness (4 questions), healthy skepticism (4 questions), and objectivity (3 questions). Answers are rated on a scale of 1 point for “not so” to 5 points for “very much so”; a higher score indicates a stronger critical thinking disposition. The Cronbach α value was 0.84 in the studies of both Yun [27] and Shin et al [28] and was 0.83 in our study.

#### Self-Efficacy

The Korean version of the General Self-Efficacy Scale developed by Schwarzer and Jerusalem [29] and adapted by Schwarzer et al [30] was used to determine general self-efficacy. The Korean version of the General Self-Efficacy Scale comprises 10 questions rated on a 4-point Likert scale ranging from 10 to 40, with higher scores indicating higher self-efficacy. The Cronbach α value was 0.90 and 0.88 in the studies by Schwarzer and Jerusalem [29] and Schwarzer et al [30] and it was 0.86 in our study.

#### Clinical Judgment Rubric

The LCJR, developed by Lasater [31], was used to evaluate the simulation experience. This rubric comprises 11 items based on the following four phases: noticing, interpreting, responding, and reflecting. The LCJR evaluates participants’ performance as beginning (1 point), developing (2 points), accomplished (3 points), or exemplary (4 points). The total score ranges from 11 to 44, with a higher score indicating higher clinical judgment ability. The Cronbach α value was 0.83 in the study of Shin et al [32] and was 0.92 in our study.

### Procedures

#### Development Overview

**Figure 2:**
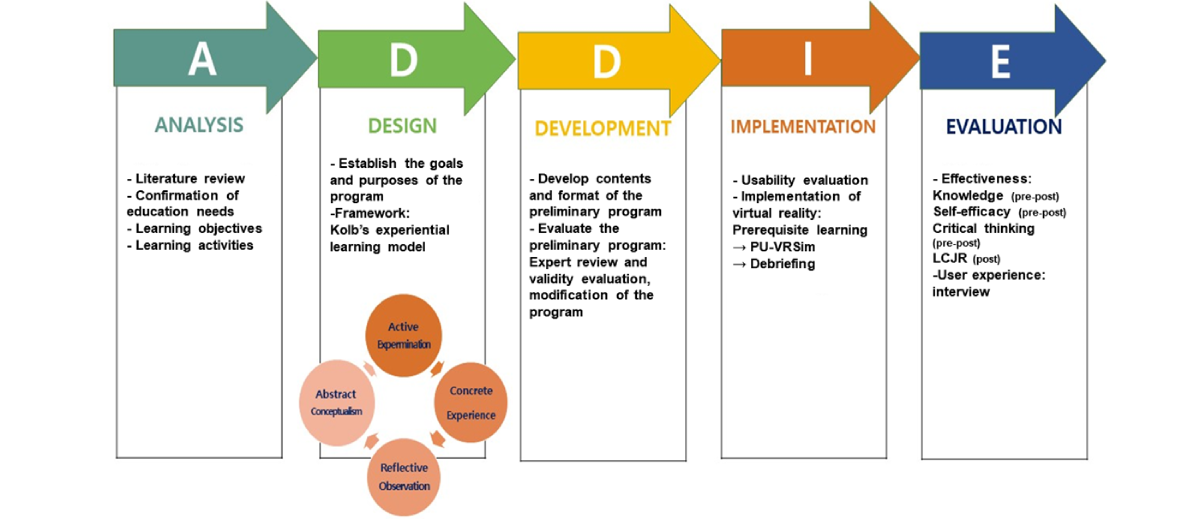
PU-VRSim program development. LCJR: Lasater Clinical Judgment Rubric; PU-VRSim: pressure ulcer management virtual reality simulation.

PU-VRSim was developed by applying Kolb’s experiential learning theory to the ADDIE model (Figure 2).

#### Analysis Stage

The analysis stage identified learners’ general and learning-related characteristics. Through a literature review [33], the importance of PU care, factors of PU occurrence, and prevention and management methods were confirmed, and the factors to be included in the development of the VR simulation were analyzed.

#### Design Stage

In the design stage, the teaching method for developing an effective educational program was determined. Kolb’s experiential learning theory [21] was applied to the basic data collected during the analysis stage to determine the teaching method using the VR simulation (concrete experience) and debriefing (reflective observation). The design aimed to improve critical thinking, self-efficacy, and clinical judgment (abstract conceptualization). Using this approach, the learner would assist in performing PU prevention and nursing (active experimentation) well in actual patients.

#### Development Stage

In the development stage, a VR-based program was developed based on the educational topics selected in the analysis and design stages. A VR platform (Unity 3D, Unity Technologies) was constructed in collaboration with a professional company. A preliminary VR program was tested by five nurses with more than 5 years of clinical experience in scenarios and nursing care of patients with PUs. By checking and correcting errors in the VR program, operational problems were improved and addressed.

#### Implementation and Evaluation Stage

The implementation stage involved application and operation of the program completed in the development stage (Table 1).

**Table 1:** Core contents and images of the virtual reality system.

Main contents			Components		Image
Login			Registration of users		
Objectives			Identifying learning objectives: Assess the degree of risk of developing PUs^a^ in the patient. Classify the PU stage of the patient. Apply a proper dressing to the patient’s PU. Provide prevention education and care for PUs to the patient.		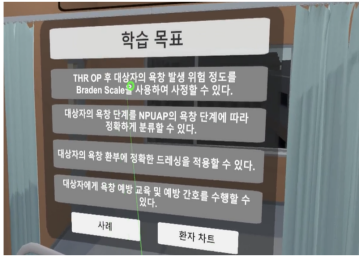
Patient case			Patient hospitalization history Patient information: name, sex, age, diagnosis, past medical history, social history		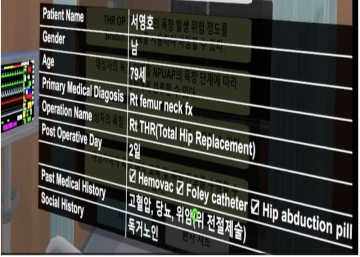
Patient information			Identifying data: vital signs, results of blood test, x-ray findings, physical exam, medication		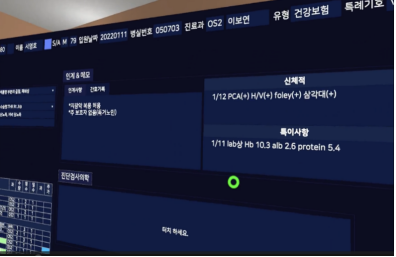
**Nursing intervention**					
	Risk assessment for PU prevention	Braden scale score; a lower score indicates a higher risk of developing PUs.		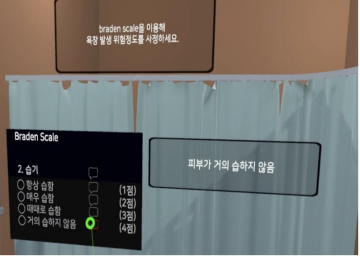	
	Assessment and evaluation of PUs	Assessment of PUs: size and stage Evaluation of PUs: writing a report		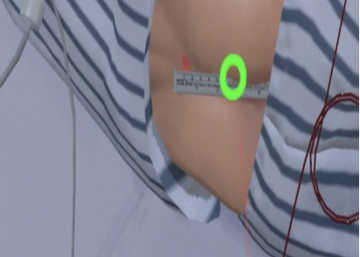	
	Management of PUs	Dressing on PUs Stage 1: film dressing Stage 2: foam dressing		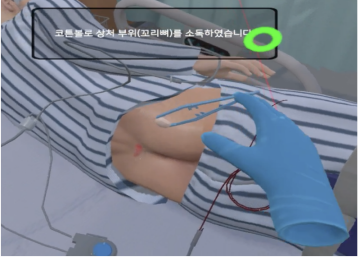	
Patient education			PU prevention and management education: Skin care, urinary and fecal incontinence management Support surfaces Repositioning Nutrition		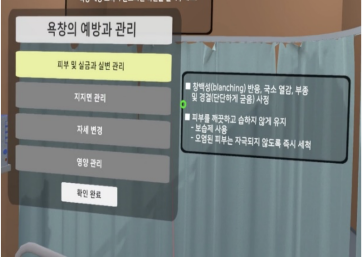
Final confirmation			Running times Feedback (recording)		

^a^PUs: Pressure ulcers

### Assessments

#### Overview of Study Design

The program was used during the evaluation stage. PU knowledge, critical thinking, and self-efficacy in the experimental and control groups were measured once before the start of the study and then again after the program. For the experimental group only, assessment using the LCJR was performed after the program and the effect of PU-VRSim was evaluated through an interview.

#### Preintervention Survey

The preintervention survey of the experimental group was conducted from October 1 to November 30, 2022, and that of the control group was conducted from January 1 to March 1, 2023. After the participants signed a consent form to participate in the study, their PU knowledge, self-efficacy, and critical thinking were measured using structured questionnaires as described above.

#### Implementation

The experimental group received the VR simulation program, comprising a prebriefing session (15 minutes) where participants briefly learned about the definition, classification, prevention, and wound management of PUs. Participants were then exposed to PU-VRSim (10 minutes), including PU assessment, nursing care, and education to patients through VR. This was followed by a debriefing session (20 minutes), in which participants were assessed using the LCJR after the simulation.

The control group received a video-based lecture. The video format was selected to reduce the time burden on participants who work in shifts and to ensure safety from SARS-CoV-2 infection, according to the participants’ hospital work. In total, 17 participants in the control group received lecture materials and a 30-minute video-based lecture on the definition, classification, prevention, and management of PUs.

#### Postintervention Survey

After the program, PU knowledge, self-efficacy, and critical thinking were assessed in both groups. The experimental group was further assessed using the LCJR and an interview was conducted to confirm their experience.

#### Interview

To discuss the experience of participating in PU-VRSim, which could not be verified using objective data, the participants were interviewed after the program. The interview included a self-introduction by the researcher and participant, recording of the interview, guaranteeing anonymity, and explaining that the research results were used only for research purposes and that the interviews were conducted with the participants’ voluntary consent. One-on-one interviews were conducted in all cases in a quiet seminar room. Before conducting the interviews, the questions were drafted based on the purpose of the study and proceeded in the order of introduction, transition, and main questions, as shown in Textbox 1.

**Interview question structure.**

Introduction question: Thank you for taking the time after work to participate in the virtual reality (VR) program. Can you briefly describe your feelings?

Transition question: Now, we would like to take the time to talk freely about the program’s effectiveness and improvements.

Main question: What helped you with the program? What do you think about the content and methods of the VR program in which you participated? What do you need to improve or add to this program?

### Data Analysis

The data collected in this study were analyzed using IBM SPSS 23.0, and a two-tailed test was performed at a significance level of .05. The normality of the dependent variable was verified using the Shapiro-Wilk test. The homogeneity of the data in the experimental and control groups was verified using the *χ*^2^ test and independent *t* test. General characteristics and performance on the LCJR aspects of the participants were presented as means (SDs) and n (%), respectively. Wilcoxon signed rank and Mann-Whitney *U* tests were used to verify differences in PU knowledge, critical thinking, and self-efficacy between the experimental and control groups.

The data collected through the interviews were analyzed using an inductive approach, which is one of the content analysis methods suggested by Elo and Kyngäs [34]. For the data analysis, the researcher repeatedly read the transcripts of the interviews, interpreted the meaning of the key statements, and created categories by assigning titles. After data analysis, the authors discussed their interpretations to reach a consensus. Subsequently, the semantic units identified were grouped into higher-level categories, the properties were stated, and the keywords were derived by coding the contents accordingly.

## Results

### Feasibility of PU-VRSim

Our first objective was to assess the feasibility of implementing the PU-VRSim program for nursing education on PUs in the implementation and evaluation stages.

The general characteristics of the participants are presented in Table 2. The average age and work experience of the 35 novice nurses was 24.8 years and 14 months, respectively. The experimental group comprised 18 (100%) women, whereas the control group comprised 2 (12%) men and 15 (88%) women. We analyzed the homogeneity of the two groups in terms of general characteristics such as age, educational level, VR experience, and PU education experience; no significant difference was observed between the two groups (all *P*>.05) and thus homogeneity between the two groups was confirmed.

**Table 2:** Characteristics of participants (N=35).

Characteristics		Experimental group (n=18)		Control group (n=17)		*χ*^2^ or *t*^a^		*P* value
Age (years), mean (SD)		24.11 (1.32)		25.53 (2.72)		–1.98		.06
**Sex, n (%)**						1.46		.16
	Male	0 (0)	2 (12)					
	Female	18 (100)	15 (88)					
Work experience (months), mean (SD)		14.06 (7.75)		14.65 (7.17)		–0.23		.82
**Education, n (%)**						1.852		.83
	College	0 (0)	3 (18)					
	University	18 (100)	14 (82)					
**PU^b^** **education, n (%)**						1.00		.33
	Yes	17 (94)	17 (100)					
	No	1 (6)	0 (0)					
**VR^c^ experience, n (%)**						0.882		.38
	Yes	3 (17)	7 (41)					
	No	15 (83)	10 (59)					

^a^*df*=17 for the experimental group and 16 for the control group.

^b^PU: pressure ulcer.

^c^VR: virtual reality.

### Effect of PU-VRSim on Outcomes

Our second objective addressed the effects of the VR intervention on PU knowledge, self-efficacy, critical thinking, and critical judgment.

As shown in Table 3, in the experimental group, the PU knowledge score increased by 2.88 points and the self-efficacy score increased by 0.56 points compared with those in the preintervention survey. In the control group, the PU knowledge score increased by 4.12 points, the critical thinking score increased by 4.0, and the self-efficacy score increased by 0.76 points. Each group showed significant improvements in PU knowledge after the intervention. However, there were no significant differences in critical thinking and self-efficacy in either group. There were no significant differences in the change in PU knowledge, critical thinking, and self-efficacy between the two groups.

The results for the clinical judgment assessment are summarized in Table 4. In the experimental group, after PU-VRSim, the overall clinical judgment of novice nurses was 3.23 points. When evaluated in the four phases to confirm whether all phases reached the level of “accomplished,” out of a total of 4 points, the mean scores for noticing, interpretation, responding, and reflecting were 3.27, 3.31, 3.32, and 2.91 points, respectively. The items “well-planned intervention/flexibility” and “skill proficiency” in the responding phase scored the highest, with 3.67 points, whereas “commitment improve” in the reflecting phase scored the lowest, with 2.78 points.

**Table 3:** Effect of the pressure ulcer management virtual reality simulation on outcomes.

Variable		Preintervention, mean (SD)	Postintervention, mean (SD)	*Z*	*P* value^a^	Difference				
						Mean (SD)	*Z*		*P* value^b^	
**Knowledge**							–0.81		.42	
	Experimental (n=18)	24.39 (4.79)	27.28 (4.43)	–3.45	.001	2.88 (2.40)				
	Control (n=17)	24.71 (5.42)	28.82 (3.41)	–2.78	.005	4.12 (4.74)				
**Critical thinking**								–1.51		.13
	Experimental (n=18)	99.00 (10.70)	98.61 (8.51)	–0.13	.896	–0.39 (5.95)				
	Control (n=17)	94.82 (7.24)	98.82 (9.36)	–1.40	.163	4.00 (9.01)				
**Self-efficacy**								–0.57		.57
	Experimental (n=18)	29.11 (3.41)	29.67 (3.24)	–1.45	.148	0.56 (1.69)				
	Control (n=17)	25.59 (4.18)	26.35 (3.98)	–1.16	.247	0.76 (3.40)				

^a^Wilcoxon signed rank test.

^b^Mann-Whitney *U* test.

**Table 4:** Clinical judgment scores.

Clinical judgment phase			Score, mean (SD)
Overall			3.23 (0.44)
**Noticing**			
	Total	3.27 (0.45)	
	Focused observation	3.17 (0.51)	
	Recognizing deviations from expected patterns	3.06 (0.54)	
	Information seeking	3.61 (0.50)	
**Interpretation**			
	Total	3.31 (0.60)	
	Prioritizing data	3.28 (0.67)	
	Making sense of data	3.33 (0.59)	
**Responding**			
	Total	3.32 (0.46)	
	Clear communication	3.11 (0.58)	
	Well-planned intervention/flexibility	3.67 (0.49)	
	Being skillful	3.17 (0.62)	
**Reflecting**			
	Total	2.91 (0.62)	
	Evaluation/self-analysis	3.06 (0.64)	
	Commitment to improvement	2.78 (0.73)	

### Qualitative Outcomes of the PU-VRSim Experience Among Novice Nurses

#### Overview of Themes

For the third objective, the interviews were grouped into five main themes: (1) realistic VR scenarios, (2) helpfulness of VR learning, (3) usability, (4) satisfaction, and (5) limitations of VR equipment (Table 5).

**Table 5:** Qualitative outcomes of the pressure ulcer (PU) management virtual reality (VR) experience in novice nurses.

Themes and subthemes		Description
**Realistic VR scenarios**		
	Real environment	There was a sense of presence in the hospital
	Real experience	Actual practice in nursing on PUs
**Helpfulness of VR learning**		
	Improving knowledge	Remember the concept and care of PUs well
	Improving skills	Perform overall practice (assess-evaluate-manage) for PUs
**Usability**		
	Safety	Safe from infection due to low contamination
	Accessibility	Able to participate freely regardless of time and place
**Satisfaction**		
	Joy and fun	It was more like playing games than learning
	New experience	It was a new and interesting learning experience
Limitations of VR equipment		Inconvenient to use equipment

#### Theme 1: Realistic VR Scenarios

The participants gained practical experience through VR scenarios. The subtopics related to this were “real field” and “real experience.”

When I experienced it myself, it was lively and felt like a real clinical environment.

S1, S8, S10, S16

It was realistic to be able to assess and manage wounds about which I learned from books using VR

S4

Although it is VR for pressure ulcer nursing, which is difficult to understand only through lectures, it was good to apply it as a direct action

S11

I only practiced with lying mannequins, but it felt more realistic when I experienced it with patients in VR like a real clinical environment.

S13

#### Theme 2: Helpfulness of VR Learning

The participants expressed that VR was helpful for learning. Subtopics related to this theme were “improvement of knowledge learning” and “improvement of skills.”

I was able to learn the stages of PUs and the types of dressings.

S6

I was able to learn about PU care.

S8

When applying nursing care to patients with PUs through VR, it seems to be more memorable.

S9, S15, S16

I was able to confirm the PU classification concept.

S12

There were no bedsore patients in the ward where I worked, and through this virtual reality program, I was able to evaluate, intervene, and evaluate PUs.

S2

I was able to perform overall nursing activities for patients with PUs.

S6

#### Theme 3: Usability

The participants expressed the feeling of using VR learning as “safe” and “easy to access.”

There were times when the mannequins were dirty in nursing practice, but it was nice that the virtual patients were not contaminated.

S13

It was nice to be able to participate without the burden of time and place.

S5

#### Theme 4: Satisfaction

The participants expressed their satisfaction of using VR learning as “pleasure,” “fun,” and “new experience.” They showed interest in VR and experienced fun and enjoyment through learning.

It feels more like playing a game than learning something. Enjoyed it.

S4

It was my first time using virtual reality, and I was able to enjoy it. I want to try again.

S5, S13, S17

It was a new experience, and I enjoyed it.

S8, S14, S17

#### Theme 5: Limitation of VR Equipment

The participants expressed limitations in terms of the equipment used in the VR program.

When I put the equipment on my head, I took off my glasses and put it on, so it was difficult to read the text because my vision was not clear.

S5

It was a bit heavy to wear on my head.

S8

The preparation for running the program was complex and took a long time.

S12

It took a long time when the focus was not good, and the text looked blurry and the controller was not recognized well in VR.

S13

## Discussion

### Principal Findings

In this study, we developed PU-VRSim by applying the ADDIE model and Kolb’s experiential learning theory [20,21]. PU-VRSim was designed as a PU prevention and nursing simulation program for novice nurses with less than 2 years of clinical experience. The participants of the PU-VRSim group showed significant improvements in PU knowledge. They reached the accomplished phase of clinical judgment. They commented that it was realistic and helpful for learning about PU management.

PU-VRSim was developed by applying an analysis-design-development-implementation-evaluation method according to the ADDIE model [20]. In the analysis stage, a literature review [33] confirmed that PU care was an important indicator of the quality of nursing services, which is becoming increasingly important [35]. PUs are caused by immobility, pressure, and friction. Factors to be included in education were analyzed by evaluating the methods for preventing and managing PUs through support surface management, position change, and dressing application to relieve pressure on the skin surface. Previous studies [36,37] have confirmed the improvement in PU knowledge and nursing performance of nurses through PU nursing education, thereby suggesting that continuous education on PUs for nurses is needed.

Kolb’s experiential learning theory [21] applied at the design stage of PU-VRSim connects theory and practice in VR simulation. Through concrete experience and reflective observation, an abstract conceptualization of theories in realistic situations can help to acquire the knowledge and skills that can be used in real situations. Kolb’s theory has also been applied in simulation education in various health fields [38], and VR provides learners with experience-based learning in a real environment by which the learners make decisions and take appropriate actions in real situations [10]. Through PU-VRSim, novice nurses freely apply the theoretical knowledge acquired through existing knowledge and prior learning materials to the process of solving problems encountered by patients in a safe virtual clinical environment. Ultimately, positive results can be expected by applying the improved nursing capabilities in actual clinical trials.

After novice nurses underwent the program, they showed improvement in PU knowledge and reached the “accomplished” stage of clinical judgment. PU knowledge scores increased on average in both the experimental and control groups after the educational program. This shows that the effect of knowledge transfer [16] can be confirmed via both traditional teaching and lectures and with the new VR simulation method. According to Kolb’s experiential learning theory, during knowledge transfer via VR, learners can improve their knowledge by reapplying it through concrete experiences and reflections, as well as learn how to utilize what they have learned and gain new knowledge. PU knowledge is the basis of PU care, and professional nursing can be performed through critical thinking and improvement of clinical performance skills. Furthermore, clinical judgment is a particularly important skill in nursing that has also received recent attention. VR has a positive impact on clinical judgment in nursing education [39]. Therefore, positive effects and acquisition of new skills can be expected if field-tailored simulation [40] is applied to nurses to reproduce clinical situations.

Interview contents were analyzed to confirm the experiences of the novice nurses participating in the PU-VRSim program. The analysis revealed that the realistic scenarios of PU-VRSim help in learning, with usability of safety, easy accessibility, and satisfaction being expressed as positive experiences. However, inconveniences in using the equipment to implement VR programs were expressed as negative experiences. This is consistent with the findings of Adhikari et al [41] on the experience of VR programs in terms of acceptability, applicability, areas of improvement, and limitations. VR can be safely and repeatedly applied in situations that can be dangerous to patients; however, it is expensive and has usage limitations [42]. VR was deemed to be a safe and effective educational method for use during the COVID-19 pandemic [43]. The development of a VR program that reduces the inconvenience and cost burden of equipment is expected to increase the use of VR in nursing education.

Our participants confirmed that PU-VRSim was helpful for learning because it could be used repeatedly to access disease-focused nursing problems. There is a need for education about various clinical situations in which nurses can apply nursing interventions according to the situation and an overall assessment of the patient [44,45]; PU-VRSim reflects the clinical situation of PUs and requires nursing education. In addition, it was confirmed as a positive experience, suggesting that improvement in nursing knowledge and clinical performance ability as well as repetitive learning are possible through the promotion of spontaneous thinking and immediate feedback of learners, which were evaluated as advantages of simulation in previous studies [46,47]. These results confirm the possibility of using PU-VRSim as an educational program in clinical practice.

In nursing practice using mannequins during the COVID-19 pandemic, the participants expressed concerns about infection via contamination of the mannequins from multiple contacts. Using VR, they felt safe as the risk of infection could be avoided. In previous studies, VR simulation was suggested and used as a nonface-to-face practice method when clinical practice was not possible due to the prevalence of COVID-19 [43,48]. In addition, participants did not feel the burden of time and space when participating in VR education. This is an advantage of VR, in which one can experience the actual medical field using only computers and equipment. Furthermore, individual learning is possible; therefore, it can provide optimal learning to individual learners and help them overcome obstacles in the physical environment [49]. VR may be an appropriate training method for shift workers, because nurses who work shifts can access the education without experiencing a time burden.

Novice nurses in this study regarded VR education as a new experience and evaluated it as enjoyable. A VR learning environment enhances immersion and activates learners’ imagination to simulate the real world [50]. The VR program is a teaching method that incorporates the latest technology and meets the learning needs of a new generation. Educational programs are being developed on various topics for nursing students and novice nurses, and the effects of enjoyment and fun have been confirmed [13]. Learning satisfaction through the enjoyable and fun VR improves learners’ learning motivation and confidence, and they experience reduced fear in real situations [14]. Because enjoyment and fun in learning are factors that stimulate learning motivation and interest, gamification can be applied when developing programs so that learners can enjoy various experiences in the virtual world.

When implementing the VR program, participants had difficulty in using an HMD; in particular, participants wearing glasses experienced inconvenience when wearing the device along with their glasses. As in previous studies, most of the participants experienced technical difficulties [41]. A VR program is typically executed using a computer program, an HMD, and a controller; however, the HMD and controller devices do not recognize the participants’ fine movements, making it difficult to proceed [42]. In the future, the development of a convenient version of the HMD, with a clear field of view, ease of wearing, and usability, may lead to an increase in the use of VR education.

PUs are common health problems in hospitals, and novice nurses experience difficulties in treating PUs. Education of nurses has been regarded as an integral component of PU prevention [51]. VR is an ideal educational technology, and the number of educational programs applying VR in nursing education has been increasing recently [52]. For the VR education program, we confirmed the improvement in knowledge of the participants through the experience in prevention and nursing interventions for patients with PUs. Improvement in clinical performance can be expected with improved knowledge. In addition, the novice nurses in this study expressed satisfaction with VR education as a new experience and a safe learning method. Considering the limitations of VR equipment, it is necessary to develop and utilize a popular simulation program that is more user-friendly and can be manipulated easily. Based on this study, we suggest the development of VR nursing education programs focusing on the educational needs of novice nurses and including new technology such as artificial intelligence with the development of technology.

### Conclusion

The PU-VRSim program developed in this study was found to be effective in improving novice nurses’ knowledge of PUs and was positively evaluated as a pleasant experience conducive to learning in an actual hospital-like environment. Therefore, PU-VRSim can be used as an effective educational method for novice nurses, as well as for nursing students and clinical nurses. In addition, a synergistic effect can be expected when the content used incorporates various software programs, including VR simulation programs.

Limitations exist in understanding and generalizing the effects of nonrandomized control-group experiments targeting novice nurses. To supplement this, we propose a follow-up study that applies the PU-VRSim program to nursing students and clinical nurses, as well as a randomized control group experimental study of novice nurses. All participants in the experimental group were women; therefore, we propose a further study with a more heterogeneous group of participants by including male and female nurses. In addition, we suggest the development of a field-tailored VR simulation for health professionals, including novice nurses, and study of its educational effect. Finally, developing a program for VR simulation is expensive and wearing an HMD when implementing the program is uncomfortable. We propose the development of software and VR simulations using technology such as smartphone apps, which are inexpensive, comfortable, and easy to use. In conclusion, we propose the continuous development and improvement of VR nursing education programs for novice nurses applying new technologies.

## Abbreviations

ADDIE: Analysis, Design, Development, Implementation, and Evaluation
HMD: head-mounted device
LCJR: Lasater Clinical Judgment Rubric
PU: pressure ulcer
PU-VRSim: pressure ulcer management virtual reality simulation
PZ-PUKT: Pieper-Zulkowski pressure ulcer knowledge test
VR: virtual reality
